# CM-LSTM Based Spectrum Sensing

**DOI:** 10.3390/s22062286

**Published:** 2022-03-16

**Authors:** Wantong Chen, Hailong Wu, Shiyu Ren

**Affiliations:** 1 Civil Aviation Flight Wide Area Surveillance and Safety Control Technology Key Laboratory, Civil Aviation University of China, Tianjin 300300, China; wtchen@cauc.edu.cn; 2 School of Electronic Information and Automation, Civil Aviation University of China, Tianjin 300300, China; 2020022132@cauc.edu.cn

**Keywords:** spectrum sensing, machine learning, long short-term memory, covariance matrix

## Abstract

This paper presents spectrum sensing as a classification problem, and uses a spectrum-sensing algorithm based on a signal covariance matrix and long short-term memory network (CM-LSTM). We jointly exploited the spatial cross-correlation of multiple signals received by the antenna array and the temporal autocorrelation of single signals; we used the long short-term memory network (LSTM), which is good at extracting temporal correlation features, as the classification model; we then input the covariance matrix of the signals received by the array into the LSTM classification model to achieve the fusion learning of spatial correlation features and temporal correlation features of the signals, thus significantly improving the performance of spectrum sensing. Simulation analysis shows that the CM-LSTM-based spectrum-sensing algorithm shows better performance compared with support vector machine (SVM), gradient boosting machine (GBM), random forest (RF), and energy detection (ED) algorithm-based spectrum-sensing algorithms for different signal-to-noise ratios (SNRs) and different numbers of secondary users (SUs). Among them, SVM is a classical machine-learning algorithm, GBM and RF are two integrated learning methods with better generalization capability, and ED is a classical, traditional, and spectrum-sensing algorithm.

## 1. Introduction

In recent years, machine learning has been widely used in the fields of speech recognition and text translation, and more and more experts and scholars have applied machine-learning algorithms to spectrum sensing to improve its performance [1,2,3]. Integrated learning algorithms in machine learning combine multiple classification algorithms to improve the accuracy of classification by combining multiple “single learners” with some strategy. For example, in [4,5], the authors use an integrated learning algorithm for spectrum sensing, which solves the problem of poor performance under low signal-to-noise ratio (SNR) conditions, and they compare this algorithm with support vector machine (SVM) and show that the integrated learning-based spectrum-sensing algorithm can achieve higher performance. In [6,7], the authors take the use of random forest (RF) for spectrum sensing, which does not require prior knowledge of the noise distribution, and a comparison with several machine-learning algorithms such as decision trees, and plain Bayes shows that the RF model has the best performance. In [8], the authors use the classical machine-learning classification algorithm SVM for spectrum sensing, which is tested using signals from real environments and compared with algorithms such as energy detection (ED); the results show that the SVM-based spectrum-sensing algorithm is more accurate than ED. Both [9,10] used temporal data of the signal as the input data for the long short-term memory network (LSTM) network model to study spectrum sensing as a classification problem, and the performance was significantly improved in the case of a single primary user (PU) and a single secondary user (SU) with superior detection performance in a colored noise background.

To improve the performance of spectrum sensing, Amr Hussein et al. used an array antenna model [11] to simulate the case of multiple SUs. The elements of the covariance matrix of multiple signals represent the spatial correlation between the signals, and the correlation is large when the PU is present, and small vice versa, thus identifying the presence or absence of the PU [12]. Cao K. and Yang Z. first applied random matrix theory [13] (RMT) in the field of wireless communication to achieve high-performance spectrum sensing. Since then, spectrum-sensing algorithms based on RMT have been widely used. The signal data processing method based on RMT has some features that the traditional data processing method does not have. These include obtaining the spatial correlation features of the signal. Many scholars form a sampled signal matrix and calculate its covariance matrix by acquiring the perceptual data of multiple SUs. The signal covariance matrix is used as the input data of the model [14,15,16]. Compared with using the energy features of the signal data, etc., this improves the performance of spectrum sensing. Examples of machine-learning algorithms based on the signal covariance matrix are as follows: in [17], the authors used SVM for spectrum sensing, using the signal covariance matrix as the input data for the model, and the used algorithm improves the detection probability over the current blind spectrum sensing (BSS) [18] algorithm in a strong noise background. In [19], the authors use the signal covariance matrix map (GSM-CM) as the input for the convolutional neural network (CNN) model, and the algorithm uses GSM-CM for spectrum sensing, which has a better performance for orthogonal frequency division multiplexing (OFDM) signals in a low SNR environment. In [20], the authors used deep neural networks to learn features autonomously from the data itself and used the sample covariance matrix as the input to the CNN model, which was modeled according to the actual environment, and the algorithm significantly improved the detection performance compared to the conventional maximum eigenvalue detection algorithm.

Based on the above machine-learning-based spectrum-sensing algorithms, we present a summary. In [5], the cyclic spectrum of the signal is used as the input of the ensemble learning algorithm to learn the cyclostationary feature of the signal. In [6], the amplitude of the signal is used as the input of the RF algorithm to learn the periodic spectrum feature of the signal. In [9,10], the temporal series data of the signal are used as the input of LSTM to learn the temporal correlation feature of the signal. In [16], the covariance matrix of the signal is used as the input of the SVM algorithm to learn the spatial feature of the signal. In [19,20], the covariance matrix of the signal is used as the input of the CNN algorithm to learn the spatial feature of the signal. In essence, the spectrum-sensing algorithm described above is used to select appropriate input features and combines with the machine-learning algorithm to obtain good sensing performance. The reason for this is that input features are one of the determinants of the performance of machine-learning algorithms [21], and the selection of appropriate input features is crucial. In addition, the above algorithm learns only a single feature of the signal in terms of energy, cyclostationarity, temporal correlation or spatial correlation.

Motivation: Based on the previous research, we expand the idea and consider that in the case of the array antenna model, multi-channel signals have spatial cross-correlation features, and single-channel signal sequences also have temporal auto-correlation features. We then choose the LSTM algorithm, which is good at extracting temporal correlation features, and take the signal covariance matrix, which reflects spatial correlation features, as its input, thus realizing the joint learning of the above two types of features. The simulations show that our CM-LSTM algorithm has excellent detection performance, which verifies that we have a CM-LSTM-based spectrum-sensing algorithm that perfectly matches the machine-learning algorithm with its input features.

Contribution: The main contributions of this paper can be summarized as follows: (1) We take the dimensionally transformed signal covariance matrix as the input of LSTM, which enables LSTM to better distinguish feature differences between signal and noise. Input features are one of the determinants of the performance of machine-learning algorithms [21], and it is crucial to choose appropriate input features. The multi-channel signal covariance matrix contains spatial cross-correlation, and the covariance matrix features of signal and noise are obviously different. The simulation results show that excellent sensing performance can be obtained by using the dimensionally transformed multi-channel signal covariance matrix as the input to the LSTM. (2) We choose the LSTM algorithm that is good at extracting temporal correlation features to learn the covariance matrix of multi-channel signals, so that the CM-LSTM algorithm can simultaneously learn the temporal correlation features of single-channel signals and the spatial correlation features of multi-channel signals. Through simulation, the chosen algorithm is compared with GBM, RF, SVM in machine learning and the classical spectrum-sensing algorithm ED algorithm in non-machine-learning algorithms. We confirm that the CM-LSTM-based spectrum-sensing algorithm has high detection performance under different SNR and different numbers of SUs. (3) We choose the array antenna model simulation to generate data, which makes the generated signal data more suitable for the actual application scenario. The array antenna model includes a single transmitting antenna and multiple receiving antennas, which are consistent with the spectrum-sensing scenario we studied with one PU and multiple SUs, and after the position of the reference antenna is determined by the array antenna model, the positions of other receiving antennas can be obtained quickly, which makes the process of generating signal data more convenient.

The rest of this paper is organized as follows. In Section 2, we present a system model for the case of multiple SUs simulated by an array antenna model. In Section 3, data preprocessing of the original signal data is presented, and the CM-LSTM spectrum-sensing algorithm and algorithm flow are detailed. Section 4 presents a simulation analysis of the CM-LSTM spectrum-sensing algorithm and compares it with three machine-learning algorithms GBM, RF, and SVM, and a non-machine-learning algorithms ED. The conclusions are discussed in Section 5.

## 2. System Model

Spectrum sensing can be expressed as a binary hypothesis test problem:

(1) $$x\left(n\right)=\left\{\begin{array}{cc} u(n), & H_{0}\\ s(n)+u(n), & H_{1} \end{array}\;\left(n=1,2,\ldots ,N\right)\right.$$

where $H_{1}$ indicates that the channel to be detected is occupied by the user, and $H_{0}$ indicates that the channel is idle, s(n) represents the transmitted signal of the PU, u(n) represents the independent identically distributed additive white Gaussian noise with a mean value of 0 and a variance of $\sigma_{n}^{2}$, and *N* is the number of sampling points of the received signal.

Assuming that the signal system model has *M* array element antennas to receive signals, the signal received by the antennas can be expressed as:

(2) $$x_{i}\left(n\right)=\left\{\begin{array}{cc} u_{i}\left(n\right), & H_{0}\\ a\left(\theta \right)s\left(n\right)+u_{i}\left(n\right), & H_{1} \end{array}\;\left(i=1,2,\ldots ,M\right)\right.$$

where θ is the space angle vector of the arrival wave, a(θ) is the direction vector.

If the first antenna is selected as the reference point, then a(θ) can be expressed as:

(3) $$a\left(\theta \right)={\left[1,e^{-jr_{2}^{T}k},e^{-jr_{3}^{I}k},e^{-jr_{4}^{T}k},\cdots ,e^{-jr_{M}{}^{T}k}\right]}^{T}$$

where $r_{i}\left(i=1,\cdots ,M;r_{1}=0\right)$ is the position vector relative to the reference point, and *k* is the wave number vector such as $k=k{\left[\cos\theta ,\sin\theta \right]}^{T}$.

After simplification, we can obtain:

(4) $$a\left(\theta \right)={\left[1,e^{-j\pi \cos\theta_{2}},e^{-j\pi \cos\theta_{3}},e^{-j\pi \cos\theta_{4}},\cdots ,e^{-j\pi \cos\theta_{M}}\right]}^{T}$$

Let:

(5) $$\begin{array}{cc} \; & V={\left[x_{1},x_{2},\cdots ,x_{M}\right]}^{T} \end{array}$$

(6) $$\begin{array}{cc} \; & u={\left[u_{1},u_{2},\cdots ,u_{M}\right]}^{T} \end{array}$$

where $u_{i}$ is the noise signal received by the i-th antenna and $x_{i}$ is the sampled signal received by the i-th antenna.

*X* is then expanded into the following matrix form:

(7) $$V=\left[\begin{array}{c} x_{1}\\ x_{2}\\ \vdots \\ x_{M} \end{array}\right]=\left[\begin{array}{cccc} x_{1}\left(1\right) & x_{1}\left(2\right) & \cdots & x_{1}\left(N\right)\\ x_{2}\left(1\right) & x_{2}\left(2\right) & \cdots & x_{2}\left(N\right)\\ \vdots & \vdots & \vdots & \vdots \\ x_{M}\left(1\right) & x_{M}\left(2\right) & \cdots & x_{M}\left(N\right) \end{array}\right]$$

The sample covariance matrix of the sampled signal is given by:

(8) $${\hat{R}}_{x}=\frac{1}{N}\sum_{k=1}^{M}x_{k}x_{k}^{H}=\frac{1}{N}VV^{H}=\left\{\begin{array}{cc} \sigma_{n}^{2}I_{M}, & H_{0}\\ R_{s}+\sigma_{n}^{2}I_{M}, & H_{1} \end{array}\right.$$

where $\sigma_{n}^{2}$ is the noise variance, $I_{M}$ represents the identity matrix of order *M*, and $R_{s}$ is the sample covariance matrix of the transmitted signal.

**Figure 1:**
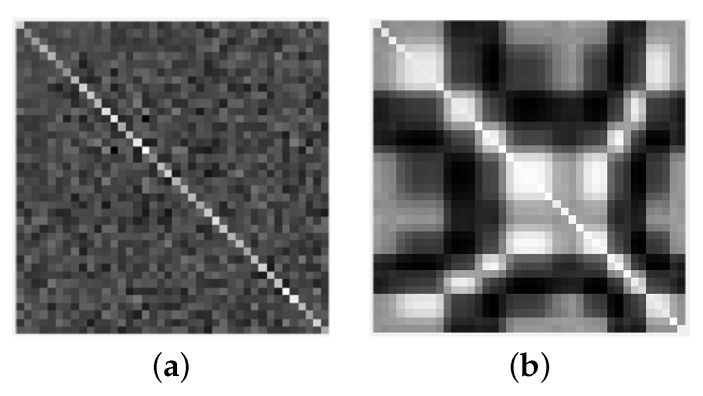
The grayscale image of the sample covariance matrix of noise and signal with 40 array element antennas: (**a**) $H_{0}$ under $R_{x}\left(N\right)$; (**b**) $H_{1}$ under $R_{x}\left(N\right)$.

Assuming there are 40 array element antennas, the grayscale images of the sample covariance matrix under the assumptions of $H_{0}$ and $H_{1}$ are shown in Figure 1a,b, respectively. When there is no PU, the values other than the diagonal are very small, and the brightness distribution of the grayscale image is relatively uniform. When a PU exists, all the values except the diagonal are larger, and the brightness distribution of the grayscale image is uneven.

The elements in the covariance matrix represent the correlation between random variables. The smaller the correlation, the smaller the element value, and the darker the brightness of the grayscale image. The diagonal elements are the autocorrelation of random variables. When the correlation is strong, the value is larger, and the diagonal brightness of the grayscale image is brighter. The off-diagonal elements represent the cross-correlation between random variables. The correlation is weaker than the autocorrelation. The off-diagonal brightness is darker than the diagonal brightness of the grayscale image. As shown in Figure 1a, the noise is random, the correlation between the noise received by different SUs is weak, and the color of the grayscale map outside the diagonal is darker; as shown in Figure 1b, different SUs receive the same transmit signal, and the useful signals received by the users are mutually correlated, and the brightness of the grayscale map outside the diagonal is brighter compared with Figure 1a.

The grayscale maps of the useful signal and the noise are distinctly different, i.e., the covariance matrix features of the useful signal and the noise are distinctly different, so we use the LSTM model to extract these features and achieve spectrum sensing.

## 3. Spectrum-Sensing Algorithm Based on CM-LSTM

The spectrum-sensing algorithm based on CM-LSTM performs spectrum sensing according to the obtained historical data information, without prior information of the PU and without determining the noise distribution. The covariance matrix after dimensional transformation is used as the input data of the LSTM model, so that the CM-LSTM algorithm can simultaneously learn the spatial correlation features of multiple signals received by the antenna array and the temporal correlation features of single signals. The CM-LSTM spectrum-sensing algorithm consists of two modules, data preprocessing, and model training and sensing.

### 3.1. Data Preprocessing

Matlab is used to generate a dataset, generate a series of random sequences at the transmitting end, and enter the Gaussian white noise channel after QPSK modulation. The array element antenna module is then used to simulate multiple SUs, so that each antenna receives different data information.

In the experiment, the SNR range is [−20 dB, 0 dB], the interval is 1dB, the numbers of array element antennas are set to 10, 20, and 40, and the number of sampling points is 1000. The collected signal is calculated to obtain the covariance matrix of the signal. After the signal covariance matrix is dimensionally transformed, the total amount of data is (2000 × 100), (2000 × 400) and (2000 × 1600), respectively. The dataset is divided by slicing. The first 700 datasets and last 700 datasets are used as training sets, while the remaining datasets are used as validation sets.

### 3.2. CM-LSTM Spectrum-Sensing Model

#### 3.2.1. LSTM Model Structure

**Figure 2:**
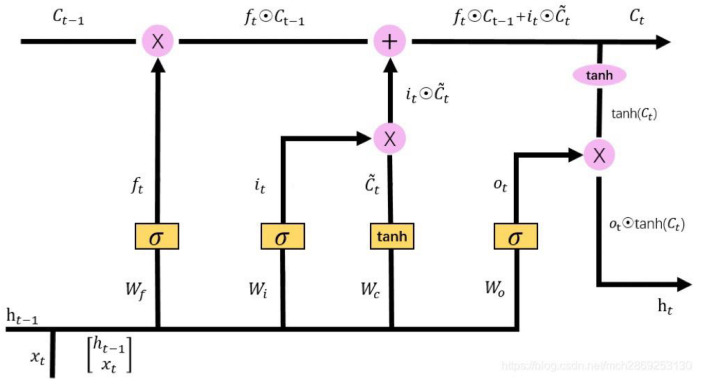
LSTM model structure.

The internal structure of the LSTM [22] model is shown in Figure 2, where $x_{t}$ is the input of the unit, $h_{t}$ is the output of the LSTM unit, $h_{t-1}$ is the output of the previous LSTM unit, $c_{t}$ and $c_{t-1}$ are the current and previous unit states, respectively, σ (from left to right) are the values of the memory gate, forget gate and output gate, respectively, ⊙ is the Hadamard product and tanh is the activation function.

The gates are used to select the information. They include a sigmoid neural network layer and a pointwise multiplication operation. The input is a vector; after the activation function, the output is a real vector between 0 and 1.

#### 3.2.2. Training and Sensing of the CM-LSTM Spectrum-Sensing Model

The dimensional transformed signal covariance matrix is transmitted to the LSTM model as the input data, so that the LSTM model can extract the spatial correlation features and temporal correlation features of signal data. The first step consists of passing through the forget gate layer, while discarding the useless information. After the activation function, the output value is between 0 and 1 and is transmitted to the memory gate. The memory gate decides to store useful information in the cell state. The sigmoid layer determines the value to be updated, and the tanh layer creates a new candidate value vector to generate the candidate memories. For the missing attribute information of the forgetting gate, the corresponding new attribute information is found in the memory layer, added to supplement the discarded attribute information, and provided to the output gate. According to the state of the cell, the sigmoid function is used to determine the part of the cell state that needs to be output. It is then processed by the tanh layer. After the two are multiplied, they pass through the fully connected layer, and the softmax classifier is used to obtain the final result and determine whether the PU exists. The flow of the spectrum-sensing algorithm based on CM-LSTM is shown in Algorithm 1, where timesteps represent the length of time series, an epoch represents all the data that are sent to the network to complete a forward calculation and back propagation process, batch_size represents the number of data samples captured in one training, num_class represents the number of categories of the model output result, $W_{f}$, $W_{i}$, $W_{c}$, and $W_{o}$ represent the weight matrix, $b_{f}$, $b_{i}$, $b_{c}$, and $b_{o}$ represent the bias term, $o_{t}$ is the output of the output gate, and $f_{t}$ is the output of the forget gate.
**Algorithm** **1** CM-LSTM spectrum-sensing algorithm.**Input**: train sample, test sample**Output**: test accuracy**Data preprocessing:**  1:  Label the data  2:  Divide the dataset in stages:  3:  (0, 700),(1000, 1700) Divided into training sets  4:  (700, 1000),(1700, 2000) Divided into validation sets**Model phase:**  5:  Input data  6:  Set up timesteps = 1, epoch = 100, batch_size = 32, num_class = 2  7:  Forget gate: choose to forget some unimportant information in the past by calculating.  8:  $f_{t}=\sigma \left(W_{f}\cdot \left[h_{t-1},x_{t}\right]+b_{f}\right)$  9:  Memory gate: decide which of the newly entered information $x_{t}$ and $h_{t-1}$ will be retained.10:  $i_{t}=\sigma \left(W_{i}\cdot \left[h_{t-1},x_{t}\right]+b_{i}\right)$11:  ${\tilde{C}}_{t}=\tanh\left(W_{C}\cdot \left[h_{t-1},x_{t}\right]+b_{C}\right)$12:  After passing the forget gate and memory gate, update the information of the next state.13:  $C_{t}=f_{t}*C_{t-1}+i_{t}*{\tilde{C}}_{t}$14:  Output gate: calculate the output of the signal through the updated information state.15:  $o_{t}=\sigma \left(W_{o}\left[h_{t-1},x_{t}\right]+b_{o}\right)$16:  $h_{t}=o_{t}*\tanh\left(C_{t}\right)$17:  Use the cross entropy loss function to calculate the loss value between the predicted value and the true value.18:  $Loss=-[y\log\hat{y}+\left(1-y\right)\log\left(1-\hat{y}\right)]$19:  Optimize the loss value through the adam optimizer until the loss converges.20:  Dense: the softmax classification function is used for classification through the dense layer.21:  **return** Enter the number of test data n, count the number of correct tests, record it as *k*, and record k/n as the obtained accuracy rate.

## 4. Experiment Analysis

In machine learning, choosing different hyperparameters, loss functions, classification functions, and optimization functions will generate different results, where hyperparameters are variables determined based on experience and the effectiveness of the validation set on the model. Therefore, we select appropriate parameters for the four algorithms GBM, RF, SVM, and CM-LSTM to ensure the fairness of the algorithm comparison, and analyze the hyperparameters and function combinations of the four machine-learning algorithms, the combination of CM-LSTM, GBM, RF, and SVM algorithms with the highest accuracy is obtained. Then, the structure of the CM-LSTM algorithm is analyzed, the role of each layer in the algorithm structure is taken, and its advantages are shown. Finally, we compare the performance of the four algorithms GBM, RF, SVM, and ED with the CM-LSTM algorithm, and verify the performance of the algorithm.

### 4.1. Algorithm-Related Parameter Analysis

#### 4.1.1. Analysis of Related Parameters of CM-LSTM Algorithm

We compared the spectrum-sensing results by training the model with different hyperparameters epoch and batch_size (cf. Table 1). At epoch 100 and batch_size 32, the spectrum detection accuracy is the highest, reaching 100%.

In addition, the spectrum-sensing results when using different classifiers, loss functions, and optimizer functions, are compared (cf. Table 1). Data transmission in the model needs to ensure that the dimensions match each other. Under the Pytorch framework, the ouput of softmax and categorical_crossentropy are compressed into two dimensions, and the outputs of sigmoid and binary_crossentropy are compressed into one dimension. Therefore, softmax and categorical_crossentropy, sigmoid and binary_crossentropy are performed as a combination. It can be seen from Table 1 that the accuracy of the combination of softmax and categorical_crossentrop is 100%, while the accuracy of the combination of sigmoid and binary_crossentropy is 98%. The accuracy difference between the two is small because the classification functions and loss functions are used for classification tasks. For the two classification tasks, the loss functions of categorical_crossentropy and binary_crossentropy are almost the same. However, since the derivatives on both sides of the sigmoid function gradually approach 0, it is easy to cause the gradient to disappear, while the softmax function avoids the disappearance of the gradient by introducing an exponential function. Therefore, the accuracy of the combination of softmax and categorical_crossentrop is higher. When using the SGD optimizer, the gradient update is frequent, which causes the loss function to highly oscillate and eventually stay at the local minimum or saddle point, resulting in an accuracy decrease. The RMSProp optimizer adds a second-order momentum on the basis of SGD, and uses the window sliding weighted average to calculate the second-order momentum, which solves the problems of local minimums and saddle points. The Adam optimizer integrates the first-order momentum of SGD and the second-order momentum of RMSProp. The first-order momentum can reduce the parameter update speed, which reduces the shock. When the gradient direction is the same, it can accelerate the parameter update, which accelerates the convergence. The second-order momentum can solve the problems of local minimum and saddle point. The Adam optimizer integrates the advantages of first-order momentum and second-order momentum, and has a strong robustness to the selection of hyperparameters. Therefore, it has a higher accuracy rate.

In summary, in order to train the model, we choose the hyperparameter combination at epoch 100, a batch_size of 32, the softmax classifier, the categorical crossentropy loss function, and the Adam optimizer.

#### 4.1.2. Analysis of Related Parameters of GBM Algorithm

In the GBM algorithm, n_estimators defines the number of decision trees that need to be used, max_depth defines the maximum depth of the tree, min_samples_leaf defines the minimum number of samples required for the end node in the tree, min_samples_split defines the minimum number of samples that a node in the tree needs to split, and max_features defines the number of features used for classification. The appropriate choice of these parameters is to prevent the model from overfitting, and the choice of these hyperparameters is a variable determined by experience and the effect of the validation set on the model. Among them, n_estimators, max_depth, min_samples_leaf, and max_features are set to a large value, which is easy to overfit, and a small set of min_samples_split is easy to overfit. Therefore, after adjusting the parameters, we obtain an accuracy of different values, as shown in Table 2. In addition, subsample is the ratio of the subsamples used to train each decision tree to the total samples; setting its value smaller than 1 can make the model more stable. According to experience, it is generally set to 0.8, and after parameter debugging, we achieve a higher accuracy when the subsample is 0.7.

In summary, we choose the parameter combination of n_estimators as 120, max_depth as 7, min_samples_leaf as 60, min_samples_split as 130, max_features as 9, and subsample as 0.7 to train the GBM model.

#### 4.1.3. Analysis of Related Parameters of RF Algorithm

In the RF algorithm, n_estimators defines the maximum number of weak learners, where, if its value is small, it easily leads to under-fitting, and, if its value is large, will lead to too much calculation. After the value of n_estimators reaches a certain number, the model improvement obtained by increasing n_estimators will be very small, so we generally choose a moderate value, which defaults to 100. However, after debugging parameters, we see that when n_estimators is 144, the model has high accuracy, and max_depth defines the maximum depth of the decision tree, which is usually 10–100. Generally, when the amount of data is small, there is no limit to its depth. However, our total data are (2000 × 100), (2000 × 400) and (2000 × 1600), and their size is moderate, so we set it to 20 through debugging, as shown in Table 3. In addition, random_state is a random seed that generates a random number every time; random seeds are very important for parameter adjustment processes, and if we use different random seeds every time, even if the parameter values are unchanged, the results will be different every time, which is not conducive to comparing the results of different models. Therefore, we set it to 0 here to ensure that, when random_state is 0, training the model with the same parameter values will return the same results.

In summary, we choose the parameter combination of n_estimators as 144, max_depth as 20 and random_state as 0 to train the RF model.

#### 4.1.4. Analysis of Related Parameters of SVM Algorithm

In the SVM algorithm, the values of hyperparameter penalty factor C and kernel function coefficient gamma are $10^{-8}$–$10^{8}$, where C represents the penalty coefficient of the model for errors, and gamma reflects the distribution of data mapped to high-dimensional feature space. Generally, the values of C and gamma are between 0.1–10, then, according to the performance of the model, we multiply them by 0.1 or 10 every time as a step size to determine the approximate range, and then refine the search interval to determine the final value. Through this debugging step, we finally determined that C is 0.5 and gamma is 0.8. In addition, because we are studying nonlinear problems, we use kernel functions to transform nonlinear problems into linear problems, in which poly is a polynomial kernel function which can map data from low-dimensional space to high-dimensional space, but there are many parameters and a large amount of computation. RBF is a Gaussian kernel function that can map samples to high-dimensional space, but compared with the polynomial kernel function, it requires fewer parameters, and it can be seen from Table 4 that the RBF kernel function has higher performance, so we chose to use the RBF kernel function. The degree and cafe0 parameters are only valid for poly kernel function, where degree denotes the highest degree of the polynomial, and cafe0 denotes the constant value of polynomial kernel function.

In summary, we choose a combination of parameters with C as 0.5, gamma as 0.8, and kernel function RBF to train the SVM model.

### 4.2. Structural Analysis of CM-LSTM Model

In order to have a clearer understanding of the chosen algorithm, its structure is presented in Table 5, where the parameters corresponding to each layer, the total number of parameters and the number of training parameters, are detailed. Among them, the LSTM layer performs feature extraction on the input data, while the batch_normalization layer normalizes the output of the previous layer, which can speed up the training and network convergence, control the gradient explosion, prevent the gradient from disappearing and efficiently prevent overfitting. The dense layer undergoes nonlinear changes to the previously extracted features, extracts the associations between these features, and maps them to the output space. Since the problem we are studying is binary classification, it can be seen from Table 5 that the final output shape of the model is 2. In addition, the number of training parameters of the chosen algorithm is of 947,586 bits, which is less than 5 Mb. It can also be seen that the training parameters occupy a small amount of memory and the equipment requirements are low, thus the chosen algorithm does not require expensive equipment, which also increases its feasibility.

### 4.3. Comparison of the CM-LSTM, GBM, RF, SVM, and ED Algorithms

The spectrum-sensing models based on GBM, RF, SVM, ED, and CM-LSTM were simulated, and we compared the accuracy of the five models under different SNR and analyzed the influence of the number of different array element antennas on the accuracy of the four models. The parameter settings of all the algorithms are presented in Table 6.

**Figure 3:**
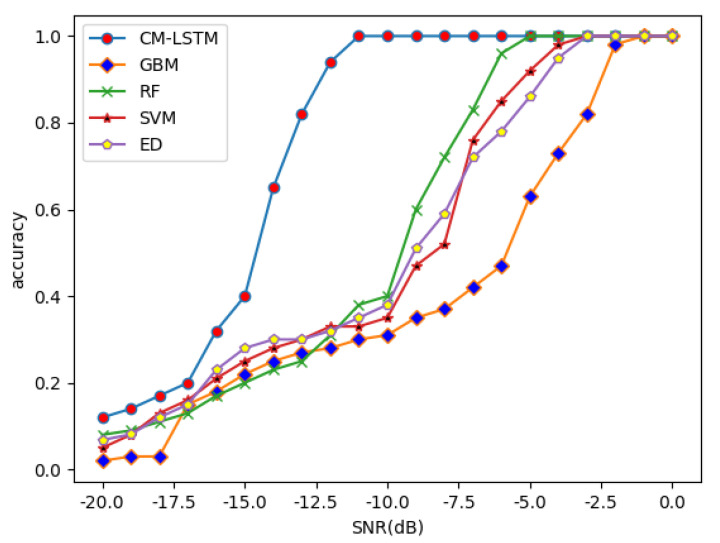
Obtained accuracy with different SNR.

In order to evaluate the performance of the chosen algorithm, at 40 antennas, the accuracy of the five algorithms of GBM, RF, SVM, ED, and CM-LSTM under different SNR is compared, as shown in Figure 3. It can be clearly seen that, in the case of low SNR and high SNR, the accuracy of the spectrum-sensing algorithm based on CM-LSTM used in this paper is better than that of GBM, RF, SVM, and ED algorithms. This is more obvious at low SNR. When the SNR is −10 dB, the accuracy of the CM-LSTM algorithm can reach 100%, while GBM and SVM are only 35%, RF is 40%, and ED is 38%. This is because the CM-LSTM spectrum-sensing algorithm can simultaneously extract the spatial cross-correlation features of multiple signals received by the antenna array and the temporal autocorrelation features of each signal compared with the other four algorithms, thus having a higher accuracy.

**Figure 4:**
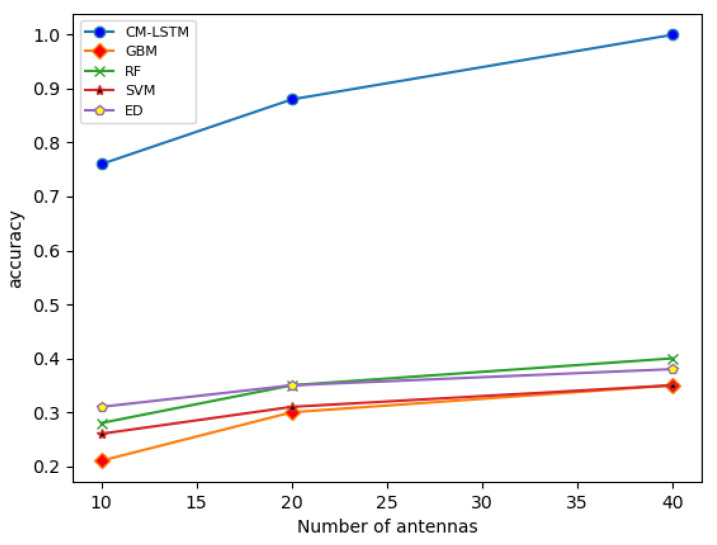
Accuracyof the different algorithms as a function of the number of antennas, for an SNR of −10 dB.

In order to further evaluate the performance of the chosen algorithm, when the SNR is −10 dB, the five algorithms of GBM, RF, SVM, ED, and CM-LSTM are simulated with different numbers of antennas (cf. Figure 4). It can be seen that the accuracy variation of all the algorithms has the same law. The amount of received signal data increases with the increase in the number of antennas. The model can perform feature extraction on more signal and noise information, making the difference in features between signal and noise more significant, thus the model has a higher accuracy rate. Moreover, the CM-LSTM algorithm has higher accuracy than GBM, RF, SVM, and ED for different numbers of antennas.

## 5. Conclusions

This paper uses machine-learning technology to study the spectrum-sensing problem as a classification problem. A CM-LSTM spectrum-sensing algorithm is studied. The algorithm can extract not only the spatial cross-correlation features of multiple signals received by the antenna array, but also the temporal autocorrelation features of single signals, which significantly improves the performance of spectrum sensing. Through simulation verification, the parameter combination that makes the CM-LSTM spectrum-sensing algorithm the most accurate is obtained. Finally, the CM-LSTM-based spectrum-sensing algorithm is compared with GBM-, RF-, SVM-, and ED-based spectrum-sensing algorithms, and the simulation analysis shows that this algorithm has better performance with different SNR and different numbers of SUs.

## Figures and Tables

**Table 1:** The accuracy of CM-LSTM model parameters and related functions.

	SNR = −10 dB	Accuracy
Epoch	50	0.92
	100	1.00
	200	0.93
Batch_size	16	0.98
	32	1.00
Classifier	sigmoid	0.98
	softmax	1.00
Loss_function	binary_crossentropy	0.98
	categorical_crossentropy	1.00
Optimizer	SGD	0.50
	Adam	1.00
	Rmsprop	0.85

**Table 2:** The accuracy of GBM model parameters and related functions.

	SNR = −10 dB	Accuracy
n_estimators	100	0.32
	120	0.35
max_depth	5	0.30
	7	0.35
min_samples_leaf	50	0.29
	60	0.35
min_samples_split	100	0.27
	130	0.35
max_features	5	0.34
	9	0.35
subsample	0.7	0.35
	0.8	0.28

**Table 3:** The accuracy of RF model parameters and related functions.

	SNR = −10 dB	Accuracy
n_estimators	120	0.36
	144	0.40
max_depth	10	0.37
	20	0.40
random_state	0	0.40
	5	0.33

**Table 4:** The accuracy of SVM model parameters and related functions.

	SNR = −10 dB	Accuracy
Kernel_function	Poly	0.31
	RBF	0.35
C	0.5	0.35
	1	0.28
gamma	0.8	0.38
	1	0.35
degree	2	0.33
	3	0.31
Cafe0	0.5	0.27
	1	0.30

**Table 5:** CM-LSTM model structure and corresponding training parameters.

Layer(Type)	Output Shape	Param
lstm_1(LSTM)	(None, 1, 128 )	885,248
batch_normalization_1	(Batch (None, 1, 128))	512
lstm_2(LSTM)	(None, 1, 64)	49,408
batch_normalization_2	(Batch (None, 1, 64))	256
lstm_3(LSTM)	(None, 1, 32)	12,416
batch_normalization_3	(Batch (None, 1, 32))	128
dense_1(Dense)	(None, 2)	66
Total parameters: 948,034 Trainable parameters: 947,586 Non-trainable parameters: 448		

**Table 6:** GBM, RF, SVM, and CM-LSTM model parameter settings.

Models	Adjusted Parameters	Value
GBM	max_depth	7
	max_features	9
	n_estimators	120
	min_samples_leaf	60
	min_samples_split	130
	subsample	0.7
RF	max_depth	20
	random_state	0
	n_estimators	144
SVM	Kernel function	RBF
	C	0.5
	gamma	0.8
	degree	3
	coef0	1
CM-LSTM	Epoch	100
	Batch_size	32
	Classier	softmax
	Loss function	categorical_crossentropy
	Optimizer	Adam

## Data Availability

Not applicable.
